# Prevalence of High Body Mass Index Among Children and Adolescents at a US Military Treatment Facility, 2008–2009

**DOI:** 10.5888/pcd9.120051

**Published:** 2012-11-15

**Authors:** Y. Sammy Choi, Cristobal Berry-Caban, Rachel Stratman, Jill H. Fleming

**Affiliations:** Affiliations: Cristobal Berry-Caban, Jill H. Fleming, Womack Army Medical Center, Fort Bragg, North Carolina; Rachel Stratman, Evans Army Community Hospital, Fort Carson, Colorado.

## Abstract

We assessed the prevalence of high body mass index (BMI) in a large cohort of military children. We compared BMI data from electronic medical records of military children aged 2 to 18 years with BMI data from the National Health and Nutrition Examination Survey (NHANES). The 23,778 military children studied were significantly less likely than the NHANES children to be overweight (27.1% vs 31.8%) or obese (11.9% vs 16.9%). Even though military parents are required to maintain fitness and weight standards, the absolute difference between military and civilian children was small.

## Objective

The prevalence of overweight in the United States is approximately 68% in adults (1) and 32% in children (2). For US military personnel, the prevalence ranges from 6.5% to 60.5% using diagnosed vs self-reported data (3,4). We assessed the prevalence of body mass index (BMI [kg/m^2^]) at the 85th percentile or higher in a large cohort of military children. We hypothesized that high BMI would be less prevalent among military than civilian children.

## Methods

Womack Army Medical Center (WAMC), Fort Bragg, North Carolina, has 6 primary care clinics that serve 117,000 enrollees, including 54,000 military members, 51,000 military family members, and 12,000 military retirees and family members. Approximately 26,000 are pediatric enrollees; more than 96% have at least 1 military parent, and less than 5% are children of retirees. Using electronic medical records (EMRs), we collected the most recent BMI data recorded between January 1, 2008, and December 31, 2009, for 36,000 children aged 2 to 18 years who received primary care exclusively through WAMC. All patients were dependents of active-duty or retired military members. We included in the sample only patients with a BMI on file.

The total eligible sample included all children enrolled during the 2-year period. Nearly 12,000 children were not included because data on height, weight, or sex were missing or because the record lacked administrative identification to match the demographic data, which was required to prevent duplication.

BMI was calculated as weight in kilograms divided by height in meters squared using EMR software. Children at or above the 95th percentile for age were categorized as obese and children between the 85th and 95th percentiles as overweight (5). We also calculated prevalence estimates for children at or above the 97th percentile. We calculated mean prevalence and 95% confidence intervals by sex and age. We assessed continuous variables for normality of distribution and compared them by using 2-tailed *t* tests. We compared categorical variables by using the χ^2^ test. Statistical significance was established at *P* < .05. We compared prevalence data for children of military parents with data that assessed children aged 2 to 19 years from the National Health and Nutrition Examination Survey (NHANES) (2). We used the *z* test for 2 proportions to determine significant differences between military and NHANES prevalence data. We used PASW Statistics for Windows, version 18.0 (SPSS, Inc, Chicago, Illinois) to conduct statistical analysis. The WAMC Institutional Review Board approved this study.

## Results

A total of 23,778 children aged 2 to 18 years was included in the study. The sample included slightly more boys (50.9%) than girls. The mean age was 8.3 years; 37.9% were aged 2 to 5, 33.9% were aged 6 to 11, and 28.2% were aged 12 to 18.

Among all children aged 2 to 18, military children were significantly less likely than children in the NHANES to be overweight (27.1% vs 31.8%) or obese (11.9% vs 16.9%) (Table 1). Except for girls aged 2 to 5 who were between the 85th and 95th percentiles, girls aged 6 to 11 who were at or above the 95th percentile, and girls aged 6 to 11 who were between the 85th and 95th percentiles, military children aged 2 to 5 and 6 to 11 were significantly less likely than the NHANES children to be overweight or obese (Table 2). However, in the group aged 12 to 18, military children were just as likely to be overweight or obese as their civilian counterparts. For all age groups, military children (both sexes combined) were less likely to be at or above the 97th percentile.

**Table 1:** Overall Prevalence of High BMI for Children and Adolescents, WAMC^a^ and NHANES^b^ (N = 23,778)

Percentile by Sex	N^c^	WAMC	NHANES	N^c^	WAMC	NHANES
	
		2–18 y			6–18 y	
	
		% (95% CI)	% (95% CI)		% (95% CI)	% (95% CI)
**≥97th percentile**								
Both sexes	1,847	7.8 (7.4–8.1)	12.3 (11.1–13.5)	1,349	9.1 (8.7–9.6)	13.0 (11.7–14.4)
Boys	1,051	8.7 (8.2–9.2)	13.9 (12.2–15.9)	757	10.1 (9.4–10.8)	14.7 (12.5–17.1)
Girls	796	6.8 (6.4–7.3)	10.5 (9.2–12.0)	592	8.2 (7.5–8.8)	11.2 (9.8–12.9)
**≥95th percentile**								
Both sexes	2,829	11.9 (11.5–12.3)	16.9 (15.4–18.4)	2,060	14.0 (13.4–14.5)	18.2 (16.5–20.1)
Boys	1,529	12.6 (12.0–13.2)	18.6 (16.4–21.0)	1,096	14.6 (13.8–15.4)	19.8 (16.9–23.1)
Girls	1,304	11.2 (10.6–11.8)	15.0 (13.3–16.8)	964	13.3 (12.5–14.1)	16.5 (14.7–18.5)
**≥85th percentile**								
Both sexes	6,455	27.1 (26.6–27.7)	31.8 (29.8–33.7)	4,443	30.1 (29.4–30.8)	33.2 (31.2–35.3)
Boys	3,371	28.9 (28.1–29.7)	33.0 (30.5–35.6)	2,275	30.3 (29.3–31.3)^d^	34.0 (30.5–37.6)^d^
Girls	3,084	26.4 (25.6–27.2)	30.4 (28.4–32.5)	2,168	29.9 (28.8–30.9)^d^	32.4 (30.0–34.8)^d^

Abbreviations: BMI, body mass index; WAMC, Womack Army Medical Center; NHANES, National Health and Nutrition Examination Survey; CI, confidence interval.

^a^ WAMC data are for 2008–2009.

^b^ NHANES data include participants aged <19 years; data are for 2009–2010 (4).

^c^ For WAMC only.

^d^ When confidence intervals overlapped, results were found to be statistically different using Z test of 2 proportions at the 0.05 level.

**Table 2:** Prevalence of High BMI by Age and Sex in Children and Adolescents, WAMC^a^ and NHANES^b^

Percentile by Sex	N^c^	WAMC	NHANES

		% (95% CI)	% (95% CI)
**Age 2–5 y**			
**≥97th percentile**			
Both sexes	498	5.5 (5.1–6.0)	9.7 (7.7–12.2)
Boys	294	6.4 (5.7–7.1)	11.5 (8.4–15.4)
Girls	204	4.6 (4.0–5.2)^d^	7.9 (5.0–12.2)^d^
**≥95th percentile**			
Both sexes	769	8.5 (8.0–9.1)	12.1 (9.9–14.8)
Boys	429	9.3 (8.5–10.2)	14.4 (11.0–18.6)
Girls	340	7.7 (6.9–8.5)^d^	9.6 (6.6–13.8)^d^
**≥85th percentile**			
Both sexes	2,012	22.3 (21.5–23.2)^d^	26.7 (22.6–31.2)^d^
Boys	1,096	23.8 (22.6–25.0)^d^	29.7 (24.0–36.1)^d^
Girls	916	20.8 (19.6–22.0)^d^	23.4 (18.5–29.2)^d^
**Age 6–11 y**			
**≥97th percentile**			
Both sexes	674	8.4 (7.8–9.0)	13.0 (11.2–15.0)
Boys	375	8.8 (8.0–9.7)	14.6 (11.9–17.9)
Girls	299	7.8 (7.0–8.7)	11.3 (9.4–13.4)
**≥95th percentile**			
Both sexes	1,033	12.8 (12.1–13.6)	18.0 (16.3–19.8)
Boys	550	13 (12.0–14.0)	20.1 (18.0–22.4)
Girls	483	12.7 (11.6–13.7)	15.7 (13.7–18.0)
**≥85th percentile**			
Both sexes	2,265	28.1 (27.1–29.1)	32.6 (30.1–35.2)
Boys	1,208	28.5 (27.1–29.8)	33.1 (30.0–36.3)
Girls	1,057	27.7 (26.3–29.1)	32.1 (28.5–35.8)
**Age 12–18 y**			
**≥97th percentile**			
Both sexes	675	10.1 (9.3–10.8)	13.0 (10.9–15.4)
Boys	382	11.7 (10.6–12.8)	14.7 (11.2–19.0)
Girls	293	8.5 (7.6–9.4)	11.2 (9.2–13.7)
**≥95th percentile**			
Both sexes	1,027	15.3 (14.4–16.2)	18.4 (15.8–21.3)
Boys	546	16.7 (15.4–18.0)	19.6 (15.2–25.0)
Girls	481	14.0 (12.8–15.1)	17.1 (14.4–20.1)
**≥85th percentile**			
Both sexes	2,178	32.5 (31.3–33.6)	33.6 (30.9–36.5)
Boys	1,067	32.6 (31.0–34.3)	34.6 (29.2–40.5)
Girls	1,111	32.3 (30.7–33.8)	32.6 (28.0–37.6)

Abbreviations: BMI, body mass index; WAMC, Womack Army Medical Center; NHANES, National Health and Nutrition Examination Survey; CI, confidence interval.

^a^ WAMC data are for 2008–2009.

^b^ NHANES data include participants aged19; data are for 2009–2010 (4).

^c^ For WAMC only.

^d^ When confidence intervals overlapped, results were found to be statistically different using Z test of 2 proportions at the 0.05 level.

Among military children, boys were more likely than girls to have a high BMI at the following age groups and percentiles: 2 to 5 years at the 97th percentile or greater (*P* < .001) and 12 to 18 years at the 97th percentile or greater (*P* < .001). For all age groups combined, boys were also more likely than girls to have a high BMI at all percentiles (*P* < .001) (Table 1).

## Discussion

Military children were significantly less likely to be overweight than their civilian counterparts. One reason could be that more than 96% of our sample has at least 1 military parent, who is required to maintain fitness and weight standards. However, the absolute difference in the prevalence of overweight between military and civilian children was small (27% vs 32%).

Limitations to this study were lack of data on parental BMI, family race/ethnicity, and socioeconomic status and the inability to identify comorbid conditions associated with high BMI in either the child or parent. Although data for 12,000 enrollees were excluded, bias was minimal because the study included 6 clinics and data on 24,000 of 36,000 eligible participants. The strength of this study was that it used a large cohort for whom BMIs calculated from measured height and weight have not been previously reported. The only other published data are a self-reported parental survey of military children aged 6 to 17 that found 16% to be overweight; 7,416 of an initial sample of 35,000 parents completed the survey (6). In our study, the prevalence of overweight in this same age group was significantly higher at 30%.

Military children accompany their parents when they are reassigned to other posts, typically every 2 to 3 years, and therefore are not substantially affected by local geography, climate, or cultural trends. Because of this circumstance and the size of our cohort, our results can be generalized to the overall military population. The prevalence of high BMI did not differ significantly among military or civilian adolescents. Because adolescent obesity is a strong predictor of adult obesity (7), intervention in the preadolescent years should be considered in civilian and military populations.
